# eHealth Literacy of German Physicians in the Pre–COVID-19 Era: Questionnaire Study

**DOI:** 10.2196/20099

**Published:** 2020-10-16

**Authors:** Johanna Kirchberg, Johannes Fritzmann, Jürgen Weitz, Ulrich Bork

**Affiliations:** 1 Department of Visceral, Thoracic, and Vascular Surgery University Hospital Carl Gustav Carus Technische Universität Dresden Dresden Germany

**Keywords:** eHealth, electronic health, mobile health, health apps, mobile health apps, eHealth literacy

## Abstract

**Background:**

Digitalization is a disruptive technology that changes the way we deliver diagnostic procedures and treatments in medicine. Different stakeholders have varying interests in and expectations of the digitalization of modern medicine. Many recent digital advances in the medical field, such as the implementation of electronic health records, telemedical services, and mobile health apps, are increasingly used by medical professionals and patients. During the current pandemic outbreak of a novel coronavirus-caused respiratory disease (COVID-19), many modern information and communication technologies (ICT) have been used to overcome the physical barriers and limitations caused by government-issued curfews and workforce shortages. Therefore, the COVID-19 pandemic has led to a surge in the usage of modern ICT in medicine. At the same time, the eHealth literacy of physicians working with these technologies has probably not improved since our study.

**Objective:**

This paper describes a representative cohort of German physicians before the COVID-19 pandemic and their eHealth literacy and attitude towards modern ICT.

**Methods:**

A structured, self-developed questionnaire about user behavior and attitudes towards eHealth applications was administered to a representative cohort of 93 German physicians.

**Results:**

Of the 93 German physicians who participated in the study, 97% (90/93) use a mobile phone. Medical apps are used by 42% (39/93). Half of the surveyed physicians (47/93, 50%) use their private mobile phones for official purposes on a daily basis. Telemedicine is part of the daily routine for more than one-third (31/93, 33%) of all participants. More than 80% (76/93, 82%) of the trial participants state that their knowledge regarding the legal aspects and data safety of medical apps and cloud computing is insufficient.

**Conclusions:**

Modern ICT is frequently used and mostly welcomed by German physicians. However, there is a tremendous lack of eHealth literacy and knowledge about the safe and secure implementation of these technologies in routine clinical practice.

## Introduction

In the health care sector, there are 2 sides to digitalization: on the one side, it offers opportunities for significant improvements; on the other side, it comes along with new and additional challenges for today’s health care system and those who work within this system [1].

Economic constraints cause health care professionals to offer their medical services at a low cost [2]. At the same time, medical treatment is increasingly personalized, individualized, and data-based [3-5]. There is also a shortage of qualified personnel in the medical field, whereas, by contrast, demographic development has led to an aging, multi-morbid society with increased medical and nursing needs and efforts. To make matters worse, the health care system is still divided into inpatient and outpatient sectors that are not adequately interconnected. The tremendous amount of medical data that is generated every day is still stored in different data silos in incompatible systems [6]. A major objective of digitalization is, therefore, to economize scarce capabilities in times of increasing workload and skill shortage in the health care business [7]. At the same time, a skill shortage of qualified and trained information and communication technologies (ICT) experts exist in the health care field [8].

Technologies with the potential to accomplish this are eHealth applications [9-11], artificial intelligence (AI) [12,13], and cloud computing [14-17]. In reality, this aim is still delayed by frequent technical problems and interface difficulties between different hardware and software systems [9]. Unfortunately, this leads to considerable frustration among health care workers [18-20]. The future aim of reducing workload by digitalization has not yet been achieved in the present intermediate stage of digitalization in the German health care system. However, several funded programs, such as the Medical Informatics Initiative, exist to overcome these problems [21].

The aim of this study is to analyze the usage of modern information and communication technologies in everyday medical practice in the pre–COVID-19 era. Furthermore, this study examines which of the possible applications are considered useful and which are considered less meaningful by physicians, and what knowledge exists regarding the technical and legal frameworks of these technologies. To our knowledge, this is the first study on this topic among German physicians. While data already exist about the specific situation of digital technology use among German physicians, specific questions of usage behavior and eHealth literacy have not yet been addressed [22]. Other international trials have surveyed the benefits and challenges arising with the usage of smartphone applications, such as medical apps or consumer messaging apps in a medical context [23-26].

All data in this trial were obtained in the pre-COVID-19 era. In our opinion, the sudden need to physically isolate doctors (for example, in tumor boards, laboratory meetings, and conferences) has massively accelerated the daily spread of digital devices and applications within the medical system [27-30]. However, our study shows that German physicians have limited or even insufficient knowledge about the safe and efficient use of eHealth technologies. Improvements in eHealth literacy are therefore urgently needed.

## Methods

A structured questionnaire with 6 categories was developed to evaluate the participants’ patterns of use and level of knowledge of eHealth applications (Multimedia Appendix 1). Furthermore, physicians’ attitudes towards the potential benefits and disadvantages of eHealth applications were analyzed. The demographic and academic characteristics of the participants were also collected anonymously. The questionnaire was developed with the help of a statistician and an expert in data management, and was tested and validated with a group of 10 persons working at the surgical trial unit in the Department of Surgery, University Hospital Dresden.

The 6 categories of the questionnaire were as follows: (1) personal characteristics (age/sex/work experience/academic degree/specialization); (2) usage profile of eHealth applications; (3) level of knowledge of eHealth applications; (4) critical evaluation of medical versus nonmedical apps; (5) critical evaluation of medical apps for patient use; and (6) critical evaluation of medical apps for physician use.

Physicians were asked to anonymously answer the questionnaire on 4 different occasions. Consent for using the anonymous data was obtained when the participants answered the questionnaire. Formal approval to conduct the surveys and to use the questionnaire was obtained from the corresponding heads of departments or conference organizers before the surveys were conducted.

The first survey was conducted during grand rounds of the Department of Visceral, Thoracic, and Vascular Surgery at the University Hospital Carl Gustav Carus, Technical University Dresden, in Germany, on August 27, 2018; of the 35 questionnaires distributed, 24 were completed. The second survey was conducted at an interdisciplinary course on pancreatic surgery for physicians from the ambulant and clinical sectors at University Hospital Carl Gustav Carus, Technical University Dresden, on August 29, 2018; of the 74 questionnaires distributed, 27 were completed. The third survey was conducted at a gastric cancer course from the Dresden School of Surgical Oncology (DSSO) on August 30, 2018; of the 8 questionnaires distributed, all 8 were completed. The fourth survey was conducted during an interdisciplinary meeting of the physicians’ association Siegen/Olpe in Kreuztal Krombach, Germany, on September 5, 2018; of the 80 questionnaires distributed, 34 were completed. The total response rate of completed questionnaires was 42% (93/197).

All data were pooled, transferred, and analyzed using Excel (version 14.0; Microsoft). Figures were created with GraphPad Prism (version 6.07; GraphPad Software Inc). Due to the limited number of questionnaires, data analysis was descriptive only.

## Results

### Participant Characteristics

Table 1 shows the participants’ demographic data. Most participants (37/93, 40%) were 30-45 years old and were men (45/93, 49 %); 29% (27/93) were women, and 22.6% (21/93) of participants did not answer the question regarding their sex. The length of working experience was 5-15 years for 31% (29/93) of participants, more than 25 years for 30% (28/93) of participants, and less than 5 years for 25% (23/93) of participants. The highest academic degrees held by the participants were state examination for 42% (39/93), a doctorate of medicine (MD) for 43% (40/93), and board certification for 63% (59/93).

**Table 1:** Demographic, professional, and academic characteristics of the study participants (n=93).

Participant characteristics		Values (%)
**Age (years)**		
	<30	17.2
	30-45	39.8
	45-60	32.3
	>60	10.8
**Gender**		
	Male	48.4
	Female	29.0
	Missing	22.6
**Working experience (years)**		
	<5	24.7
	5-15	31.2
	15-25	14.0
	>25	30.1
**Highest academic degree**		
	State examination	41.9
	Medical doctor (MD)	43.0
	Habilitation (lecturer)	4.3
	Professorship	4.3
	Missing	6.5
**Board-certified specialist**		
	Yes	63.4
	No	33.3
	Missing	3.2

### Usage Profile of eHealth Applications

The participants used eHealth applications in daily life via their mobile phones. Among the 93 participants, 90 (96.8%) had their own mobile phone, which was a smartphone in more than 90% of cases. The system software was Android for 48% (45/93) of participants and Apple/iOs for 44% (41/93), while 3% (3/93) used other software. Of the 93 physicians, 67 (72%) carried their private mobile phone with them during work; only 30% (28/93) had an official mobile phone provided by their employer (Table 2).

**Table 2:** Profile of professional mobile phone usage among study participants (n=93).

Questions regarding mobile phone usage	Responses (%)		
	Yes	No	Missing data
Do you use and own a mobile phone?	96.8	3.2	0.0
Do you use your own mobile phone during work?	72.0	28.0	0.0
Do you use a separate professional mobile phone?	30.1	65.6	4.3
Is your mobile phone a smartphone?	92.5	6.5	1.1

In general, the participants’ mobile phones were used (professionally and privately) for phone calls (89/93, 96%), messaging (eg, WhatsApp; 84/93, 90%), surfing the internet (83/93, 89%), navigation (77/93, 83%), online banking and financial issues (33/93, 36%), social media (29/93, 31%), and gaming (16/93, 17%). These results are displayed in Table 3.

**Table 3:** Purpose of mobile phone use among study participants (n=93).

Purpose of mobile phone use	Values (%)
As a telephone and for SMS text-messaging services	95.7
Messaging apps	90.3
Social media	31.2
Surfing the internet	89.2
Route planner/navigation	82.8
Online banking/finance	35.5
Medical apps	41.9
Gaming	17.2
Other	7.5

Medical apps were used by 42% (39/93) of participants. Of the 93 surveyed physicians, 50% (47/93) used their private mobile phone for official purposes on a daily basis, 24% (22/93) did so once per week, 10% (9/93) did so once per month, and 15% (14/93) never did so. Telemedicine was part of the daily routine for 33% (31/93) of all participants, while 65% (60/93) did not use it. A fitness bracelet was used by 12% (11/93) of participants.

### Critical Evaluation of Medical Versus Nonmedical Apps

To obtain further insights into the interviewees’ attitudes, the participants were asked to rank their personal levels of importance for safety, quality, fun, and design factors in medical versus nonmedical apps. For professionally used medical apps, 63% (59/93) of participants ranked data safety as most important, while 66% (61/93) ranked quality of content as most important. The fun factor was of minor importance (ie, unimportant) to 67% (62/93) of the physicians, as was the fact that family or friends used the same app (47/93, 51%). Saving time was seen as important for 66% (61/93) of participants (Table 4). For nonmedical privately used apps, 65% (60/93) of the participating physicians ranked data safety, and 62% (58/93) ranked the quality of content, as most important. Additionally, saving time (56/93, 60%) and design factors (49/93, 53%) were very important in the participants’ perceptions. The fun factor was the least important for 42% (39/93) of participants, while 41% (38/93) stated that family or friends using the same app was important (Table 4).

**Table 4:** Evaluations of the importance of professionally used medical apps and privately used nonmedical apps among study participants (n=93).

Factor		Evaluation of importance (%)				
		Not important	Important	Very important	Not applicable	Missing data
**Professionally used medical apps**						
	Design and operation	11.8	26.9	44.1	12.9	4.3
	Time saving	1.1	17.2	65.6	12.9	3.2
	Data safety	2.2	15.1	63.4	15.1	4.3
	Friends/family use the same app	50.5	11.8	9.7	21.5	6.5
	Image of provider	43.0	25.8	11.8	15.1	4.3
	Quality and topicality of contents	3.2	12.9	65.6	14.0	4.3
	Fun factor	66.7	6.5	3.2	18.3	5.4
**Privately used nonmedical apps**						
	Design and operation	5.4	35.5	52.7	5.4	1.1
	Time saving	0.0	31.2	60.2	5.4	3.2
	Data safety	3.2	24.7	64.5	5.4	2.2
	Friends/family use the same app	24.7	40.9	23.7	8.6	2.2
	Image of provider	58.1	24.7	4.3	9.7	3.2
	Quality and topicality of contents	0.0	29.0	62.4	6.5	2.2
	Fun factor	32.3	41.9	12.9	8.6	4.3

### Level of Knowledge of eHealth Applications and Data Safety

Of the 93 participants, 66% (61/93) believed that less than 30% of all apps in established app stores conformed with basic standards for data safety and safe communication; 22% (20/93) thought that at least 30-60% of apps met these standards. Furthermore, 39% (36/93) appraised messaging apps (eg, WhatsApp) as appropriate for professional communication while 53% (49/93) did not, and 89% (83/93) did not know of alternative safe messaging apps (eg, Siilo, Careflow Connect, MedCrowd). The participants who rated email communication as sufficiently safe for professional communication in the health care business were 28% (26/93), while 62% (58/93) did not agree. More than 80% of the respondents had never been asked by patients about medical apps. The findings suggest that insecurity with regard to issues of data safety is common: 82% (76/93) admitted that their knowledge was insufficient regarding the legal aspects and data safety of medical apps and cloud computing in clinical life, while 65% (60/93) held this belief about their knowledge regarding technical aspects. Furthermore, 85% (79/93) of participants thought it was necessary to perform a legally bound certification of medical apps regarding data safety, and 79% (73/93) felt this was necessary with regard to the quality of content (Table 5).

**Table 5:** Questions on the application, state of knowledge, data safety, and legal obligations of medical eHealth apps (n=93).

Question		Responses (%)			
		No	Yes	Unknown	Missing data
Is it appropriate to use common messaging apps for professional communication (eg, WhatsApp)?		52.7	38.7	8.6	0.0
Do you know of safe messaging apps for professional communication (eg, Siilo, Careflow Connect, MedCrowd)?		89.2	10.8	0.0	0.0
Is it appropriate to use common email for professional communication in health systems?		62.4	28.0	8.6	1.1
Do you have sufficient knowledge of the technical aspects of medical apps and cloud computing to evaluate their application in clinical daily work life?		64.5	21.5	12.9	1.1
Do you have sufficient knowledge of the technical aspects of medical apps and cloud computing regarding legal aspects and data safety to evaluate their application in clinical daily work life?		81.7	4.3	14.0	0.0
Do you use telemedical applications in your clinical daily work life?		64.5	33.3	2.2	0.0
Have you been asked about medical apps by patients yet?		82.8	16.1	1.1	0.0
**Do you think a legal obligation for external certification of medical apps is required** **?**					
	Regarding safe communication and data storage?	4.3	84.9	9.7	1.1
	Regarding medical effectiveness and quality of contents?	5.4	78.5	14.0	2.2

### Critical Evaluation of Medical Apps for Patient Use

The use of medication apps, coaching apps for medical issues, online scheduling apps, and emergency apps for patients was ranked as a possible idea or good idea by 86% (80/93), 82% (76/93), 81% (75/93), and 76% (71/93) of participants, respectively. Video consultation apps and follow-up apps were considered a bad idea by 39% (36/93) and 20% (19/93) of all participants, respectively, whereas 55% (51/93) and 73% (68/93) considered both to be possible and good ideas, respectively (Table 6).

**Table 6:** Physician evaluation of medical apps for patients and physicians among study participants (n=93).

Medical apps			Physician evaluation (%)						
			Bad idea		Feasible		Good idea		Missing data
**Medical apps for patients**									
	Emergency app	15.1		36.6		39.8		8.6	
	Online appointment app	12.9		50.5		30.1		6.5	
	Coaching app on illnesses	9.7		37.6		44.1		8.6	
	Video consultation app/digital health assistance app	38.7		12.9		41.9		6.5	
	Follow-up app (eg, postoperative, malignancy aftercare)	20.4		40.9		32.3		6.5	
	Medication app	8.6		58.1		28.0		5.4	
**Medical apps for physicians**									
	Diagnostic/differential diagnosis app	3.2		53.8		39.8		3.2	
	Guideline app	1.1		76.3		19.4		3.2	
	Drug app	1.1		80.6		15.1		3.2	
	Documentation/ward round assistance app	9.7		36.6		50.5		3.2	
	Digital patient record	18.3		38.7		40.9		2.2	

### Critical Evaluation of Medical Apps for Physician Use

The attitude of the participants towards medical apps for physicians differed. Large medical apps that might facilitate the physicians’ daily life ranked positively, such as guideline apps, medication apps, diagnostic help apps, and documentation help apps, which rated as possible ideas or good ideas by 96% (89/93), 96% (89/93), 94% (87/93), and 87% (81/93) of the physicians, respectively. Digital patient records were viewed more critically: 80% (74/93) of the physicians believed they were a possible idea (36/93, 39%) or a good idea (38/93,41%), whereas 18% (16/93) thought they were a bad idea (Table 6).

## Discussion

### Principal Findings

The market for health apps is confusing and difficult for individuals to grasp. There is a risk of misuse of health data related to patients. Consequently, applications in the eHealth sector must be subject to the same regulations as those otherwise provided in the health sector. Several initiatives have published evaluation guidelines for the systematic assessment of the quality of apps. Thus far, however, no uniform seal or proof of safety and quality exists [31-34]. In fact, only 30% of all commercially distributed health apps have privacy policies. Of these privacy policies, two-thirds are unrelated to the health app itself but only address commercial rights, distribution rights, or the rights of third parties. Health apps still frequently share data with third parties without the user's knowledge, often without encryption [35].

This situation is also related to the third trend in the telehealth sector: medical care for chronically ill patients is increasingly shifting from hospitals to the outpatient sector. With the help of portable diagnostic technologies coupled with smartphones and a telemedical connection of the patient to hospitals, diseases can be treated at home. Offering health services in nursing homes or at home via smartphones would follow a trend that has existed for years in other areas (eg, online shopping, online banking).

The trial population of this study was a representative cohort of German physicians in terms of age and academic degrees. There was an underrepresentation of 29% of female physicians in this study (46.8% of physicians in Germany are female), and the field “sex” was left unanswered by 22.6 % of participants due to an unfavorable layout in the questionnaire [36,37]. Certain facts reduce the general transferability of the study. The cohort mainly consisted of surgeons in the hospital sector because 3 of the 4 time points of inquiry were surgical meetings. Nonsurgical physicians of the ambulant, nonacademic sector are underrepresented. Physicians with a positive attitude towards digitalization in public health might be overrepresented. The questionnaire was self-designed, and to our knowledge, no standardized questionnaire on the topic of digitalization and eHealth exists. The sample size of 93 physicians is too small to guarantee generalizability. However, the answer rate of 47% was in the expected range for this type of survey.

All data in this trial were obtained in the pre–COVID-19 era. In our opinion, the sudden need for physical distancing (for example, in tumor boards, laboratory meetings, and conferences) has massively accelerated the daily spread of digitalization within the medical system [30]. This phenomenon may have a lasting positive impact on doctors' and patients’ attitudes towards eHealth applications and should be the subject of further study. In addition, further studies with larger sample sizes are needed.

The participants had contact with eHealth applications, mainly via their private mobile phones. In contrast to the general population in Germany, they favored Apple software (41/93, 44%) at a relevantly higher percentage. The German average of Apple users is only 20% [38,39]. The participants who used telemedicine in their professional lives were 33% (31/93), while 65% (60/93) did not use it. Most physicians (77/93, 83%) had never received questions from patients about health apps as part of their professional activities, but 16% (15/93) said they had received such questions. There is an increasing desire and need for advice among patients [40]. Furthermore, there is little knowledge among medical staff within health care facilities regarding information technology (IT) security. Great harm can be caused by unsuspecting, reckless behavior. For example, most hacker attacks in the health sector can be traced to the inadvertent installation of malware, for example, by opening file attachments in emails or using external USB sticks [41].

Almost 40% (37/93) of those surveyed considered it generally acceptable to use normal messaging apps (eg, WhatsApp) for professional communication. Only slightly more than half of the respondents considered this to be unjustifiable, although, in a previous question, 80% (74/93) of the participants considered data protection and security to be important or very important. Secure messaging apps that have been specially developed for professional communication in the medical sector, such as Siilo, Careflow connect, or Medcrowd, were only known to approximately 10% (9/93) of those surveyed. At least 30% (28/93) of those surveyed considered professional communication in the health sector via normal (unencrypted) email to be sufficiently secure and reliable, while approximately 60% (56/93) did not consider this to be sufficiently safe and reliable. This means that despite the sensitive data, unsuitable and insecure communication channels such as normal messaging apps and unencrypted email communication are still regarded by many physicians as acceptable means of communication [42,43].

The self-assessment of knowledge was remarkable. With regard to apps and cloud computing, only 20% (19/93) of the surveyed participants stated that they were sufficiently familiar with the technical aspects. With regard to legal aspects and data safety, only 4% (4/93) believed that they were sufficiently informed to assess their use. In the case of health apps for patients to intervene in diagnostics and therapy (eg, medication app), almost half of the physicians surveyed believed that these should be classified as medical devices. The survey results also show that there is little specialist knowledge among the survey participants in this area, since apps for intervention in diagnostics and therapy must be approved as medical devices in Germany.

The evaluation of medical apps for patients is also interesting. It is noticeable that apps for patients were rated more critically by the physicians than potential apps for physicians. One reason for this skepticism among physicians could be the changed doctor-patient relationship or a new way of evaluating and using medical services that would result from these applications [44,45].

The results show that there is no uniform opinion among the physicians surveyed. There seems to be a non-negligible group of up to 40% (37/93) of the surveyed physicians who are critical of the new application options. On the other hand, a large group of the surveyed physicians considered many of these applications to be feasible or potentially good ideas.

The evaluation of medical apps for physicians, however, shows a slightly different picture. Only 3% (3/93) considered an app for assistance with diagnostics and differential diagnoses to be a bad idea, guidelines were considered good by almost all of the respondents, and a drug app and a documentation and visit assistance app were considered favorable by more than 90% of the respondents. Apps for help with diagnostics, differential diagnoses, guideline display, medication, and documentation ward round assistance were rated as good ideas by up to 50% of respondents, and as feasible by up to 50% [43,46,47].

It is striking that an app for electronic medical records (EMR) was considered by almost 20% to be a bad idea, by nearly 40% to be feasible, and by approximately 40% to be a good idea. Again, there is a difference of opinion within the medical profession [48]. This is also in contrast to data from other countries, which are much more in favor of EMR [1].

Similar to the negative attitude towards some patient apps, this may also be an indicator that there are reservations in the medical profession regarding a health system that is changing through eHealth applications, which will also affect the professional profile of physicians and in which the digital patient file is one key player.

### Conclusion

Modern information and communication technologies, such as smartphones, are already often used. A large proportion of physicians are open to such new technology-based applications and are in favor of their introduction and use in practice.

The possibility of using these technologies to avoid errors in treatment, to reduce the administrative burden, and to improve supply in the area is apparent. However, a non-negligible proportion of physicians are skeptical about these applications and critically assess or reject their use, especially if the application affects the doctor-patient relationship.

There is also a considerable lack of knowledge about the application of these technologies, which leads to incorrect assessments on the part of medical professionals regarding data security and data protection. It can be assumed that modern information and communication technologies will be used in the German health care system in the near future. Due to the increasing importance of these technologies, at least basic knowledge of eHealth applications in the health sector should be provided by further training measures and by including this content in medical curricula. Digitalization in health care is advancing. The speed of its development and its acceptance by those involved will largely depend on the extent to which those affected participate in this process. Active participation is, therefore, essential for the medical profession and is the only way to advance development in patient-centered, holistic, personalized medicine. Care must also be taken to ensure that the special doctor-patient relationship is not lost.

The rapidly accelerated spread of eHealth applications within the medical system by COVID-19 protection measures since March 2020 may induce a massive and long-lasting rethinking of digitalization in the health care sector by medical professionals and trigger a boom in eHealth applications. This current development should be further studied.

## Appendix

Multimedia Appendix 1 Questionaire - Supplementary material.

## Abbreviations

EMR: electronic medical record
ICT: information and communication technologies
