# Supplementary material for: eHealth Literacy of German Physicians in the Pre–COVID-19 Era: Questionnaire Study
Source: JMIR Mhealth Uhealth. 2020 Oct 16;8(10):e20099. doi: 10.2196/20099 (PMC7600010; doi:10.2196/20099)
Supplement: Multimedia Appendix 1 [file mhealth_v8i10e20099_app1.pdf]

University Hospital Carl Gustav Carus

Clinic and Polyclinic for Visceral, Thoracic and Vascular Surgery

Clinic director: Prof. Dr. Jürgen Weitz

University Hospital Carl Gustav Carus - Fetscherstr. 74 - 01307 Dresden

Dear Colleague

New information and communication technologies (ICT), such as smartphones, apps and cloud services, are changing our private and professional everyday life forever. ICT applications, such as health apps on the smartphone, are also being used more and more frequently in the healthcare sector.

At the same time, there are many ambiguities related to the use of these technologies and applications, in particular regarding the reliability of information, security and privacy aspects.

The purpose of this survey is to get an overview of the actual use by physicians, the existing knowledge about these technologies and the evaluation of possible practical applications.

Answering the questions should not take more than 5-10 minutes. We would like to thank you very much for your assistance by filling in the anonymous questionnaire.

Your
Ulrich Bork

If you have any further questions about the project, I am available for you questions:

PD Dr. Ulrich Bork
University Hospital Dresden

Data protection notice:

This survey is anonymous. This means that at no time will your personal data, such as name, date of birth, etc. be revealed. The survey is multi-centered and the evaluation is carried out for all locations together, i.e. it is not possible to draw conclusions about individual persons even by assigning the personal data. Data collected in the course of this study will be recorded and further evaluated anonymously.

Personal details

Your age (in years) and sex:

- ☐ < 30 ☐ 30 - 45 ☐ male
- ☐ 45 - 60 ☐ > 60 ☐ female

length of professional activity (in years):

- ☐ < 5 ☐ 5 - 15
- ☐ 15 – 25 ☐ > 25

Highest academic degree:

- ☐ State Examination ☐ PhD
- ☐ Dr. (M.D.) ☐ professor

Specialist board certification:

- ☐ yes ☐ no

Questions about mobile phone (in the following mobile phone) and smartphone:

Do you use your own private mobile phone?

- ☐ yes ☐ no

Do you usually carry your mobile phone with you at work?

- ☐ yes ☐ no

Do you use an official mobile phone (provided by your employer)?

- ☐ yes ☐ no

How often do you also use your private mobile phone for business purposes (e.g. call service, apps)?

- ☐ never ☐ monthly ☐ weekly ☐ daily

Is your mobile phone a smartphone?

- ☐ yes ☐ no ☐ I don’t know

What operating system does your smartphone use?

- ☐ Apple (iOS) ☐ Android ☐ Other ☐ I do not know / not applicable

Do you have/use a fitness wristband or similar "connected device":

- ☐ No
- ☐ Fitness bracelet
- ☐ other connected device (e.g. Smart Watch)

What do you use your mobile phone / smartphone for (multiple answers possible):

- ☐ Phone calls, SMS
- ☐ Messenger (e.g. WhatsApp)
- ☐ Social media (e.g. Facebook)
- ☐ web browsing
- ☐ Route planner / navigation (e.g. Google Maps)
- ☐ Online Banking / Finances
- ☐ Medical tips / Health apps
- ☐ Games
- ☐ Other: ______________________________

Questions about the use of apps (if no use please tick "not applicable")

Which of the points are important to you for privately used apps?

	Not important	important	Very important	Not applicable
Design and usability	☐	☐	☐	☐
Time saving through use	☐	☐	☐	☐
Privacy / Security	☐	☐	☐	☐
Friends / families use the same app	☐	☐	☐	☐
Image of the manufacturer / supplier	☐	☐	☐	☐
Quality and topicality of the contents	☐	☐	☐	☐
Fun factor	☐	☐	☐	☐

Which of the points are important to you for apps used for business purposes?

	Not important	important	Very important	Not applicable
Design and usability	☐	☐	☐	☐
Time saving through use	☐	☐	☐	☐
Privacy / Security	☐	☐	☐	☐
Friends / families use the same app	☐	☐	☐	☐
Image of the manufacturer / supplier	☐	☐	☐	☐
Quality and topicality of the contents	☐	☐	☐	☐
Fun factor	☐	☐	☐	☐

Do you use medical apps in the context of your everyday work?

☐ yes ☐ no ☐ I don't know

How do you rate the following medical apps / applications for patients?

	Good idea	possible	Bad idea
1. Emergency App (e.g. by a panel doctor on call)	☐	☐	☐
2. Online appointment allocation / appointment coordination app	☐	☐	☐
3. Coaching / training on diseases	☐	☐	☐
4. Video consultation / digital health assistant	☐	☐	☐
5. Aftercare app (e.g. postoperative or for malignancies)	☐	☐	☐
6. Medication app (e.g. insulin regimen)	☐	☐	☐

How do you rate the following medical apps / applications for doctors?

	Good idea	possible	Bad idea
1. Assistance Diagnostics / differential diagnosis	☐	☐	☐
2. Guideline presentation	☐	☐	☐
3. Digital patient file	☐	☐	☐
4. Drug application (e.g. yellow list, embryotox)	☐	☐	☐
5. Documentation / visit assistance	☐	☐	☐

General questions on information and communication technology (ICT)

Do you think it is basically justifiable to use normal messenger apps (e.g. WhatsApp) for professional communication?

☐ yes ☐ no ☐ I don't know

Are you familiar with secure messenger apps (e.g. Siilo, Careflow Connect, MedCrowd) for professional communication?

☐ Yes ☐ no

Do you think cloud computing based technologies for professional communication / data exchange make sense?

☐ Yes ☐ no ☐ I don't know

Do you consider professional communication in the health sector via normal email to be sufficiently secure and reliable?

☐ Yes ☐ no ☐ I don't know

Would you say that you are sufficiently familiar with the <u>technical side of</u> apps and cloud computing to assess their use in everyday clinical practice?

☐ Yes ☐ No ☐ I don't know

Would you say that you are sufficiently familiar with the <u>legal aspects and data protection requirements</u> of apps and cloud computing to assess their use in everyday clinical practice?

☐ Yes ☐ no ☐ I don't know

Do you use telemedical applications (e.g. external diagnostic reports) in your professional activities?

☐ Yes ☐ no ☐ I don't know

Have you already received queries from patients about health advice or comparable applications in the course of your professional activities?

☐ Yes ☐ No ☐ I don't know

If a health app for patients interferes with diagnostics and therapy (e.g. contraception app, insulin scheme app), do you think it should be approved as a medical device?

☐ Yes ☐ No ☐ I don't know

In your opinion, what percentage of the apps that can currently be downloaded from the popular App Stores meet at least adequate basic data protection standards for secure communication and data storage?

☐ < 30 % ☐ 30-60% ☐ >60%

Do you consider mandatory certification by an independent third party for health care applications / medical ICT applications to be necessary?

<u>A) With regard to security and data protection</u>

☐ Yes ☐ No ☐ I don't know

<u>B) In terms of medical efficacy/quality of content</u>

☐ Yes ☐ No ☐ I don't know

Your comments (free text):

____

____
